# Effectiveness comparisons of acupuncture treatments for Bell palsy in adults: A protocol for systematic review and Bayesian network meta-analysis

**DOI:** 10.1097/MD.0000000000020252

**Published:** 2020-06-05

**Authors:** Bing Li, Xiqing Sun, Jun Guo, Wenjie Shu, Yiran Cheng, Jie Li

**Affiliations:** a First College of Clinical Medicine, Shandong University of Traditional Chinese Medicine; b Department of Neurology, Affiliated Hospital of Shandong University of Traditional Chinese Medicine, Jinan, Shandong Province; c The Key Laboratory of Geriatrics, Beijing Institute of Geriatrics, Beijing Hospital, National Center of Gerontology, National Health Commission, Institute of Geriatric Medicine, Chinese Academy of Medical Sciences, PR China.

**Keywords:** acupuncture, Bell palsy, network meta-analysis, protocol

## Abstract

**Background:**

Bell palsy (BP) is a simple peripheral facial paralysis. A variety of acupuncture treatments have been reported effective for the recovery of BP. However, the relative effectiveness of these acupuncture treatments is still unclear. Therefore, we plan to summarize the evidence and determine the most effective acupuncture treatment for BP.

**Methods:**

We will search the following database, including The Cochrane Library, PubMed, Web of Science, EMBASE, China BioMedical Literature (CBM),China National Knowledge Infrastructure (CNKI), Chinese Scientific Journals Database (VIP), and Wanfang database, from their inceptions to April 30, 2020, in order to collect randomized controlled trials (RCTs) on acupuncture in the treatment of BP. We will use Stata16.0 and WinBUGS software for statistical analysis and draw surface under the cumulative ranking curve (SUCRA) graph for each outcome indicator to predict the order of curative effect of treatment measures.

**Results:**

This study will compare and rank the effectiveness of different acupuncture methods in the treatment of BP, and the outcome indicators will include House-Brackmann Grading Scale, sequelae, Facial Disability Index score, Sunnybrook facial grading system, Portmann score, and adverse events.

**Conclusion:**

Our study will provide supports for clinical practice.

INPLASY registration number: INPLASY202040019.

## 1 Introduction

Bell palsy (BP), also known as idiopathic facial palsy or facial neuritis, is an idiopathic facial weakness or paralysis originating from the peripheral nerve, with acute onset.^[1]^ BP affects people of all ages and genders, with incidence rates ranging from 20 to 30 per 10,0000 population years, and 1 out of every 60 individuals will be affected during their lifetime.^[1]^ Study shows that 71% of BP patients return to normal facial function while 29% leave symptoms of semi-facial weakness, in which is severe and disfiguring in more than half of these cases.^[2,3]^ Besides, 1 third of patients yield anxiety and depression, and delay recognition of emotional facial expressions.^[4,5]^ Studies have shown that BP can increase the risk of cardio-cerebrovascular disease, depression, anxiety disorder, and so on.^[6–8]^ It has a significant incidence of recurrences, which not only affects the physical and mental health of patients, but also affects the social economy.^[9]^

The cause of BP is unclear and may vary from individual to individual.^[10]^ A variety of causes have been proposed, including viruses, inflammation, autoimmunity, blood vessels, and so on. Corticosteroid, antiviral, neurotrophic and vitamin B drugs are mainly used in clinical treatment.^[11–13]^ However, the use of drugs is prone to many side effects. For example, corticosteroid drugs, such as prednisone, can induce or aggravate peptic ulcer, cause malignant hypertension or lead to liver and kidney insufficiency. Antiviral drugs can cause digestive symptoms such as nausea, vomiting, and diarrhea, as well as nervous system symptoms such as dizziness and convulsions (more common with higher doses).^[14]^ The side effects of drug abuse prompt patients to seek non-pharmacological treatment. Therefore, the development of effective alternative therapy for BP is a top priority.

Acupuncture is an important therapy of traditional Chinese medicine, which means that under the guidance of the theory of traditional Chinese medicine, the needle is pierced into the patient according to a certain acupuncture angle, and stimulate a specific part of the human body through a certain manipulation, so as to achieve a certain therapeutic effect.^[15]^ There will generate a sour, numb radiating, distending pain in the needling site, which is usually called “de qi”.^[16]^ This is because acupuncture is pierced to acupoints, which are located in important meridians or meridian junctions, or on the walls of vein vessels or connective tissue junctions and so on. These special acupoints are generally rich in nerves and blood vessels, so they will produce the feeling of sour, numb radiating and distending pain. Acupuncture achieves therapeutic effect by balancing disorders in the body. Such balance is achieved by activating the bodys accurate meridians and acupoints using diverse acupuncture techniques, according to disease and personal condition.^[17]^

Acupuncture has a long history with remarkable clinical effect. In recent years, the controlled clinical trials of acupuncture in the treatment of BP have increased and there are several related systematic reviews so far^[18–21]^ Chen et al,^[18]^ Kim et al,^[19]^ and Zhang et al^[21]^ analyzed the relevant literature before May 2010, December 2010, and July 2016, respectively, and did not draw a clear conclusion that acupuncture is effective in the treatment of BP. Li et al^[20]^ searched the literature before 2015 for meta-analysis, and found that acupuncture seems to be an effective method for the treatment of BP. Zhang et al^[21]^ searched the literature before July 2018 for meta-analysis, this latest systematic review has confirmed that acupuncture is related to improving the cure rate and total effective rate of BP. Acupuncture methods are diverse, however, most literatures only consider the effectiveness of single or combined acupuncture methods on BP, meanwhile, previous systematic reviews have considered all acupuncture methods as a whole, as a result, different acupuncture methods are ignored. The relative effectiveness among different types of acupuncture for BP are not yet clear, so clinicians can only choose different acupuncture treatments according to their personal experience, sometimes they may be confused about what kind of acupuncture methods to choose.

The traditional systematic reviews and meta-analysis methods can only evaluate 2 kinds of interventions, but fail to compare interventions more than 2 kinds. With the continuous development of medicine, there are many kinds of treatment measures for the same disease. The relative effectiveness and safety of these treatment measures are the cornerstone of clinical decision-making. When there is no direct comparative evidence between these interventions, in order to find the most effective or safest intervention, the use of traditional meta-analysis is obviously stretched. Network meta-analysis (NMA) developed in recent years can solve this kind of problem very well, which enables the comparison of the efficacy of 3 or more interventions at the same time. Its biggest advantage is that the different interventions of similar diseases can be summarized for quantitative statistical analysis and comparison. Therefore, in this study, we will break the overall concept, further explore different acupuncture methods, consequently, we will conduct NMA for different acupuncture methods to examine the their effectiveness in the treatment of BP and ranked according to effectiveness, in order to help patients and clinicians make clinical choices among various acupuncture methods and provide scientific reference for clinic.

## 2 Methods

We will use the Preferred Reporting Items for Systematic Reviews and Meta-Analysis Protocols (PRISMA-P) to conduct this study.^[22]^

### 2.1 Study registration

This NMA has been registered on the International Platform of Registered Systematic Review and Meta-analysis Protocols (INPLASY) and the registration number is INPLASY202040019 (URL = https://inplasy.com/inplasy-2020-4-0019/).

### 2.2 Eligibility criteria

#### 2.2.1 Type of study

We will include all clinical randomized controlled trials (RCTs) of acupuncture in the therapy of BP in adults, regardless of whether or not use blind methods. Non-RCT or semi-RCT trials, non-clinical trials or trials that lack relevant outcome indicators will be excluded.

#### 2.2.2 Participants

We will include patients more than18 years old with BP, the diagnostic criteria will be based on the definition by American Academy of Otolaryngology-Head and Neck Surgery (AAO-HNS)^[23]^ or any other recognized diagnostic guidelines. Gender, race, nationality, and duration will not be restricted.

#### 2.2.3 Interventions and comparators

**Figure 1:**
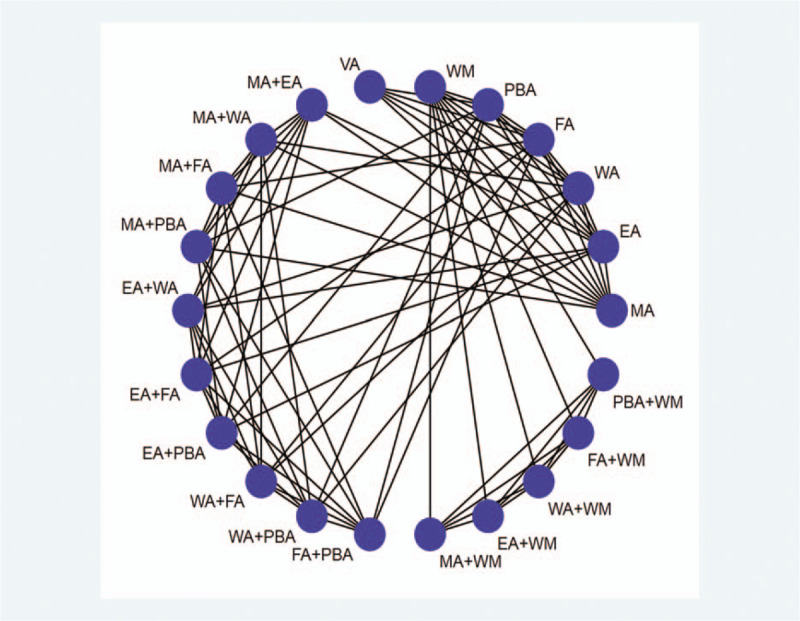
Network plot of all possible direct comparisons (MA, manual acupuncture; EA, electroacupuncture; WA = warm acupuncture, FA = fire acupuncture, PBA = plum blossom acupuncture, WM = western medicine, VA = virtual acupuncture).

The interventions of the experimental group will include manual acupuncture, electroacupuncture, warm acupuncture, fire acupuncture, plum blossom acupuncture, used alone or combination of any 2 methods, or in combination with western medicine, which must be the same as the control group. The principles related to acupuncture will be limited to traditional Chinese medicine, so non-traditional laser acupuncture, thread-embedding, bee venom acupuncture, and unrelated methods such as acupoint application and bloodletting therapy will be excluded. There will be no special requirements for acupuncture manipulation, frequency and duration. The control group will include virtual acupuncture, western medicine or other acupuncture method different from the experimental group, and other interventions will be excluded. Figure 1 shows the network plot of all direct comparisons that may be used.

#### 2.2.4 Outcomes

The primary outcome will be the recovery of facial muscles defined by House-Brackmann Grading Scale (HBGS), which classifies the facial nerve function into 6 levels (normal, mild, moderate, moderate to severe, severe, and complete dysfunction), it is also a scale of 1 to 6 points, of which 6 represents complete paralysis. The advantage of the evaluation system is that the classification method is simple and convenient, it can be widely used and includes a variety of symptoms such as mouth-eye linkage and hemifacial spasm.

The Secondary outcomes will include:

1. sequelae (including hemifacial spasm, joint movement, and crocodile tear syndrome);
2. Facial Disability Index (FDI)score, mainly conducts questionnaire surveys for patients. The body function (FDIP) and social life function (FDIS) associated with facial nerves and facial muscles will be evaluated by asking the patient's condition, which is a subjective self-assessment of the patient;
3. Sunnybrook facial grading system(SFGS), the system combines static, dynamic and joint movements of facial muscles to evaluate facial nerve function, from 0 to 100, of which 0 indicates the most severe paralysis and 100 indicates normal;
4. Portmann score, divided into 2 items, movement and quiet score, the movement mainly includes frown, close eyes, moving nose, whistling, smile, drumming cheeks, 3 points for each with a total of 18 points, plus the quiet score of 2 points, the total score is 20 points;
5. adverse events.

### 2.3 Search strategy

We will search the English and Chinese databases: The Cochrane Library, PubMed, Web of Science, EMBASE, CBM, CNKI, VIP, and Wanfang database. In addition, we will search data from literature reviews and meta-analysis and we will also search ongoing trial registers in the trial registry websites. If multiple systematic reviews of the same topic are found, we will use the latest version for the evaluation. The search date will be from their inceptions to April 30, 2020. The search strategy will be constructed in the form of Medical Subject Headings (MeSH) combine with keywords, including “bell palsy, idiopathic facial palsy, facial neuritis, acupuncture, electroacupuncture, warm acupuncture, fire acupuncture, plum blossom acupuncture, humans, randomized controlled trial”, etc. Taking Pubmed as an example, the search strategy is shown in Table 1.

**Table 1:**
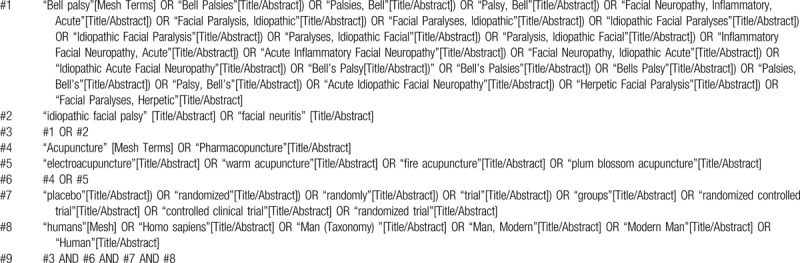
Search strategy for PubMed.

### 2.4 Study selection and data extraction

**Figure 2:**
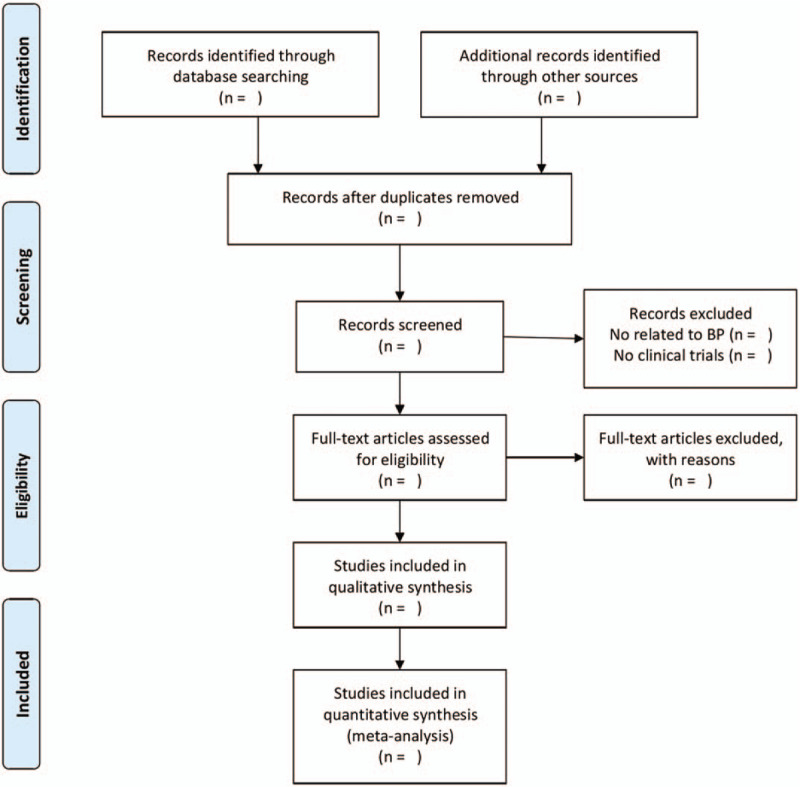
PRISMA flow chart.

We will import the documents extracted from the database into EndNoteX8, and check them manually and automatically to eliminate duplicate documents. Two reviewers will read the title and abstract of the literature independently, initially eliminate the inconsistent study, and then consult the full text of the remaining literature and eliminate the literature that is repeatedly published or does not meet the inclusion criteria, and make a record of the reasons for the deletion of the literature. Next, 2 reviewers will sort out the literature that meets the requirements and extract data independently, then cross-checked. If there are differences, discuss or consult a third party to help resolve. Two reviewers will extract the required data according to the table designed in advance and use Microsoft Excel 2016 software to record the data completely. The information extracted from the materials will include: title, author, journal, country, date of publication; Number of cases, race, disease statues, gender, age, diagnostic criteria, disease status, interventions, outcome indicators. We will extract the total number and the number of events for the dichotomous variable and extract the total sample size, mean difference (MD), and standard deviation (SD) for the continuous variable. If the SD value cannot be obtained in the original text, we will convert it by standard error (SE) or 95% confidence interval (CI). If MD is not provided in the full text, we will calculate it by the treatment baseline and the final measurement. If the report fails to provide the information required by the reviewer, contact the main author by email or phone to obtain relevant information. The results of the same clinical trial in different periods, or other forms of repeated reports, will be included as 1 clinical trial report. Figure 2 shows a PRISMA flow chart of the selection process.

### 2.5 Risk of bias assessment

The quality will be assessed by 2 reviewers independently with reference to the Cochrane Collaboration Risk of Bias Tool.^[24]^ Seven items will be evaluated, each with 3 grades namely “high”, “unclear” and “low”. In the process of evaluation, if there are disagreements, consult a third party to resolve.

### 2.6 Statistical analysis

#### 2.6.1 Pairwise meta-analysis

We will use Stata16.0 software to conduct the pairwise meta- analysis. The odds ratio (OR) will be used as the effect index for dichotomous variable while the MD used as the effect index in the continuous variable. 95% CI will be given for each effect index. We will use Cochrane Q Test (*χ*^2^) to analyze the heterogeneity between the studies and use *I*^2^ to evaluate the size of the heterogeneity.

#### 2.6.2 Network meta-analysis

Stata16.0 software will be used to map the network plot for the comparison of interventions for each outcome index. We will use WinBUGS1.4.3 software to analyze the data, using the Bayesian Markov Chain Monte Carlo (MCMC) random effect model. We will use 1 chain to simulate, the number of iterations will be set to 50 million times, in which the first 20 million times for annealing to eliminate the effect of the initial value while the last 30 million times for sampling. We will evaluate the convergence of the iteration by the Brooks-Gelman Rubin method, when the potential scale reduction factor (PSRF) tends to 1, the degree of convergence is satisfactory. In the actual operation, we will adjust the iterations and annealing times. We will choose OR as the dichotomous variable and MD as the continuous variable, and calculate the 95% CI. The difference will be statistically significant when the 95% CI of OR value does not contain 1 or the MD value does not contain 0. We will draw a SUCRA for each outcome indicator to predict the order of curative effect of treatment measures, the larger the area under the curve, the better the treatment measures.

#### 2.6.3 Assessment of heterogeneity

If *P* > .10 and *I*^2^ < 50%), fixed-effects model will be used; if there is a large heterogeneity between the research results(*P* < .1 and *I*^2^ > 50%), the source of heterogeneity will be further explored, and if the source cannot be found, random-effects model will be used for analysis.

### 2.7 Assessment of the similarity and transitivity

At present, there is no recognized statistical test method for the evaluation of similarity and transitivity. We will evaluate different studies based on clinical and methodological characteristics.^[16]^ It should be noted that all the characteristics that affect the test effect must be similar.

#### 2.7.1 Assessment of inconsistency

When there is a closed loop, we will use the node splitting method to evaluate the consistency between direct comparison and indirect comparison, or the consistency between indirect comparisons of different paths.^[25]^ When *P* < .05, the difference of consistency is statistically significant, and the consistency of the model is poor.

#### 2.7.2 Subgroup analysis and sensitivity analysis

If there is heterogeneity, we will analyze the causes of heterogeneity and conduct subgroup treatment according to different sources of heterogeneity. If it is caused by methodological quality, we will analyze it according to the quality level; if it is caused by different design schemes, we will conduct subgroup analysis according to the design scheme, country, year of publication, age, time of onset and duration can be used for subgroup analysis. We will use the exclusion method to analyze the sensitivity of all outcome indicators. If we find that heterogeneity changes with the exclusion of a certain article, then this article is the source of heterogeneity. It can be analyzed from the aspects of experimental design, sample size, outcome index, evaluation standard, and so on. If the heterogeneity remains unchanged, the result is robust.

#### 2.7.3 Assessment of publication bias

For articles with a study number greater than 10, we will construct comparison-correction funnel plots for the outcome indicators, if the funnel plot is symmetrical, there is no obvious publication bias; if the funnel plot is asymmetric on both sides, it is suggested that there may be publication bias.

#### 2.7.4 Grading the quality of evidence

We will use Grading of Recommendations Assessment, Development and Evaluation (GRADE) to evaluate the quality of evidence from the following 5 aspects: risk of bias, indirectness, inconsistency, imprecision, and publication bias.^[26]^

#### 2.7.5 Ethics and dissemination

This study does not involve personal and human trial data and therefore does not require ethical approval. We intend to publish the final results of this study in peer-reviewed scientific journals, as well as in conferences and in the mass media.

## 3 Discussion

BP is a common facial acute inflammation with sudden onset. It is often found in the morning when there are facial paralysis symptoms, which occur more unilaterally, and some occur on both sides, more common in young adults. It brings great pain to the body and mind of the patient and seriously affects the quality of life.

At present, acupuncture treatment, including manual acupuncture, electroacupuncture, warm acupuncture, fire acupuncture, plum blossom acupuncture, has been used to improve the symptoms of patients with BP. The latest meta-analysis has confirmed the effectiveness of acupuncture treatments. So far, no NMA has been performed to evaluate the comparative efficacy and acceptability of these available therapies. Therefore, it is necessary to use NMA to analyze the comparative effects of these acupuncture therapies. As far as we know, this is the first time that NMA has been used to investigate the acupuncture treatment of BP. Compared with the paired meta-analysis, NMA can analyze the treatment grades of different acupuncture methods in the analysis and evaluation of HBGS, sequelae, FDI, SFGS, Portmannscore and adverse events. In NMA, according to the area of SUCRA, we can more intuitively know the specific differences in the therapeutic effect of various treatment schemes, which is more in line with the needs of clinical application. Although NMA has some advantages that pairwise meta-analysis cannot replace, there may be some limitations in the use of NMA in our actual operation. Firstly, the frequency, duration, and other details of acupuncture will not be taken into account, which may produce heterogeneity to a certain extent. Secondly, we will only collect Chinese and English databases, but do not collect databases in other languages, which may lead to language bias. Thirdly, our research will be based on literature, not on personal data, which may cause some deviation to the results.

Nevertheless, our study is expected to rank different acupuncture methods to help clinicians and patients to choose the best treatment for BP. In addition, we hope that our results can provide support for clinical practice. It is also hoped that more large-scale randomized controlled trials will be included in the analysis and the quality of evidence-based medicine will continue to be improved, so as to provide a better basis for clinical work.

## Author contributions

**Conceptualization:** Bing Li, Xiqing Sun; Jun Guo.

**Formal analysis:** Xiqing Sun, Jun Guo, Wenjie Shu.

**Methodology:** Yiran Cheng, Jie Li.

**Project administration:** Wenjie Shu.

**Software:** Wenjie Shu, Yiran Cheng, Jie Li.

**Writing – original draft:** Bing Li.

**Writing – review & editing:** Xiqing Sun.
